# Validation of the Rowlands Universal Dementia Assessment Scale (RUDAS) to detect major neurocognitive disorder among elderly people in Ethiopia, 2020

**DOI:** 10.1371/journal.pone.0262483

**Published:** 2022-01-20

**Authors:** Beniam Daniel, Liyew Agenagnew, Abdulhalik Workicho, Mubarek Abera

**Affiliations:** 1 Department of Psychiatry, Institute of Health, Jimma University, Jimma, Ethiopia; 2 Department of Epidemiology, Institute of Health, Jimma University, Jimma, Ethiopia; Universita degli Studi Magna Graecia di Catanzaro, ITALY

## Abstract

**Background:**

The Rowland Universal Dementia Assessment Scale (RUDAS) is currently widely used for research and clinical purposes in many countries. However, its applicability and validity have not been evaluated in the Ethiopian context so far. Therefore, we designed this study to assess the reliability and validity of Rowland Universal Dementia Assessment Scale to detect major neurocognitive disorder among older people in Ethiopia.

**Methods:**

An institution-based cross-sectional study was conducted among selected older people residing in Macedonia institutional care center, Addis Ababa, Ethiopia. The gold standard diagnosis was determined using the Diagnostic and Statistical Manual of Mental Disorders criteria for major neurocognitive disorders. Stata v16 statistical software was used for data analysis. Receivers operating curve analysis, correlations, linear regression, and independent t-test were performed with statistically significant associations declared at a p-value of <0.05. Inter-rater, internal consistency reliabilities, content, criterion and construct validities were also determined.

**Results:**

A total of 116 individuals participated in the study with a 100% response rate. Most (52.7%) of the participants were male and the mean age in years was 69.9± 8. The Cronbach’s alpha for RUDAS was 0.7 with an intra-class correlation coefficient value of 0.9. RUDAS has an area under the receivers operating curve of 0.87 with an optimal cutoff value of ≤ 22. At this cutoff point, RUDAS has sensitivity and specificity of 92.3 and 75.3 with positive and negative likelihood ratios as well as positive and negative predictive values of 3.7, 0.1, 65.5%, and 91.5%, respectively. There has also been a significant difference in the mean scores of RUDAS among the two diagnostic groups showing good construct validity.

**Conclusion:**

The Rowland Universal Dementia Assessment Scale has been demonstrated to be a valid and reliable tool to detect major neurocognitive disorder. Policy makers and professionals can incorporate the tool in clinical and research practices in developing countries.

## Introduction

As the world population is going through the demographic transition, the proportion of older people is substantially increasing [1–3]. According to a 2018 report from the United Nations department of economic and social affairs, the number of people aged 65 years and above exceeded the number of under-five children for the first time in human history [1]. The proportion of older people is increasing at a faster rate in low- and middle-income countries (LMIC) than in high-income countries [1, 2, 4]. This population aging will have several social, economic, and health consequences [3]. Among the problems facing this population group more commonly are cognitive disorders [5].

Dementia or major neurocognitive disorder can be described as a syndrome in which there is a progressive deterioration in multiple areas of cognitive functioning [5–7]. According to the data from the world Alzheimer’s report 2018, 50 million people are estimated to live with dementia, and this number is projected to reach 152 million in 2050. Currently, the estimation indicates that there are new cases of dementia every 3 seconds globally [8–10].

Even though an accurate diagnosis of cognitive impairment requires a detailed and multidisciplinary assessment of the individual, many short and brief screening tools have been developed and are being used over the past several years. The availability of brief and effective screening and cognitive assessment tools is necessary especially in low- and middle-income countries where there is a recognized gap in the availability of professionals with a specialty to diagnose and provide appropriate interventions [6, 9]. These tools will contribute to the early identification of those with cognitive impairment at early stages so that available pharmacologic and non-pharmacologic interventions aimed at improving their cognitive function and quality of life can be provided before getting worse [6, 11–13].

Many of the available cognitive assessment tools were developed in western countries for the more educated and less culturally diverse population [13–15]. To this end, the effectiveness and applicability of most of these tools in communities with a very high illiteracy rate, low socioeconomic status, and more ethno-cultural diversity have been under question [14, 16–19].

The scarcity of culturally and linguistically adapted and valid cognitive screening tools in Ethiopia had made it difficult for clinicians and researchers to effectively screen and diagnose cognitive impairment [20, 21].

Ethiopia is known for its large linguistic and cultural diversity and where almost half (48.23%) of its adult population and more than 80% of those aged 65 and above are illiterate. Therefore, finding alternative cognitive assessment tools and further assessing the ones with known educational and linguistic biases is of the essence [20, 22].

Australian researchers developed RUDAS in an effort to produce a simplified tool to identify dementia that can be applied in diverse cultures, portable, and can be easily administered by primary health care providers [23–27]. RUDAS has been validated in both high-income and LMIC with demonstrated good validity and reliability and was shown to be relatively free from linguistic and educational biases [14, 28–32].

Even though many cognitive assessment tools including RUDAS have been validated for assessing cognitive impairments worldwide, the tools are culture, language, and context-sensitive and warrant the need for validation before using them in a new setting. Therefore, this study was designed with the objective of determining the psychometric properties and diagnostic accuracy of RUDAS among older people in Ethiopia to detect major neurocognitive disorder.

## Methods

The study was conducted in an institutional care centre for older people in Addis Ababa, the capital city of Ethiopia. Macedonia is an indigenous care centre for the elderly and people with mental disabilities, which is an independent, non-governmental, and a non for profit organization. Accommodation, catering, and other services are provided within the centre. The study was conducted between 10^th^ August and 15^th^ September 2020. An institution-based cross-sectional study design was employed. All individuals who were 60 years of age and older and residing within the center were included. Individuals with severe life threatening illnesses were excluded from the study.

### Sample size and sampling method

MedCalc Version 19.1.3 was used to calculate the sample size based on the assumptions and statistical methods suggested by Hanley and McNeil for determining the diagnostic accuracy of a diagnostic test or AUC [33]. Type I error (alpha) was set at 0.05, type two error (beta, 1- Power) at 0.1, i.e. Power set at 90%, the ratio between the positive and negative groups 1:2, and the null value at 0.5. The expected AUC was set at 0.7 considering the optimal AUC value for a good diagnostic test. Based on these assumptions, the total minimum required sample size was 105. After adding a 10% non-response rate, the final sample size became 116.

After generating a sampling frame from the list of individuals in the centre that fulfill the eligibility criteria, simple random sampling was employed to select study participants using the computer-generated random numbers method.

### Data collection instruments and procedures

#### A. Socio-demographic variables

A brief questionnaire was prepared to collect information on the socio-demographic characteristics of the participants including age, sex, marital status, religion, ethnicity, area of residence, and educational status.

#### B. Gold standard reference

DSM 5 criteria for dementia was used to diagnose dementia [7]. The criteria require a decline from the previously developed level of cognitive functioning that interferes with independence in daily living and that other causes for impairment be excluded. As part of the gold standard diagnostic evaluation, the MMSE was used as a standardized cognitive assessment instrument (Criteria A2) [7]. Criteria B requires the interference of the impairment with the performance of daily living activities, which include instrumental activities of daily living. Criteria D requires other conditions causing cognitive impairment to be ruled out and the geriatric depression scale was used to assess the presence of depression and rule out pseudo-dementia.

The MMSE was used as a standard cognitive assessment tool under the DSM assessment. DSM criteria A2 requires the cognitive decline to be evidenced with at least one standardized cognitive measurement scale. It is a 30-point cognitive test initially developed in 1975. to assess cognitive function [7, 15]. Modifications have been made to the original version of the MMSE as the serial sevens test for assessing attention was replaced with backward naming of the months of the year.

Depression was assessed using the Geriatrics depression scale (GDS) short form. The tool was developed by Yesavage et al. It is a 15-item self-report measure developed to examine depressive symptoms among older adults. The scale has 15 Yes/No questions [34, 35].

#### C. Rowland Universal Dementia Rating Scale (RUDAS)

RUDAS is a six-item cognitive assessment tool scored from 30 points. A cutoff value of 23 has been recommended in the initial validation study to screen for cognitive impairment. It takes less than 10 minutes to administer [23]. The six items in the RUDAS and their respective share of points are described as follows. Memory was assessed with four-item grocery list recall test in which the respondents are told a list of grocery items at the beginning of the assessment and are asked to recall them at the end (8 points, 2 for each item). Visuospatial ability is assessed in the tool through own body orientation in which the interviewer asks the respondent to show/locate different parts of his own body (5 points). Praxis is assessed with a fist/palm alternation test. The interviewer demonstrates the alternating movements of fist and palm and asks the respondent to repeat those movements continuously (2 points). The tool also has another item to assess visuo-constructional ability with the cube copying test (3 points). The respondents are then given a scenario of road crossing and their answers are used to assess their judgment (4 points). The last item is the assessment of language, which is measured by a one-minute animal generation test. The respondents are asked to generate names of as many animals as they can within one minute time. (8 points) [19, 23]. A further detailed explanation of the tool and the items can be found elsewhere [23].

#### Data collection procedures

The data was collected by two BSc nurses and two BSc psychiatry professionals. Trained BSc nurses conducted the interviews with RUDAS then the same cases were interviewed by the BSc psychiatry professionals based on the DSM approach. The order of the two tests was interchanged for every case to avoid the order of tests effect.

The two groups of data collectors, the ones applying the gold standard and those applying the test tool to be validated were blinded to the client’s performance in the other test.

In addition to the detailed clinical evaluation, the respondent’s medical record was reviewed with their permission to assist in diagnosis.

*Inter-rater reliability*. The inter-rater reliability was assessed by applying the questionnaire on 20 individuals by two data collectors blinded to the findings of one another. The same two data collectors involved in the data collection for criterion-related validity were used.

All questionnaires were translated to the Amharic language with translation and back-translation procedure. The original English version of the tool was forward translated into the Amharic language by two bilinguals proficient in both languages. The forward-translated tools were back-translated into English by another two bilingual university instructors with MSc level training who have experience translating research questionnaires. The original, the forward-, and back-translated versions of the questionnaires were reviewed and discussed upon among the team of translators involved, the principal investigator (PI), and senior psychiatry professionals. Any discrepancies were resolved after discussion with the involved professionals.

Overall, the RUDAS items were translated into the Amharic language without significant problems, and the tool showed good semantic equivalence. Some modifications were made during the translation process and are summarized below.

In all the items of RUDAS, the semantic equivalence of the term "I want you", which is *"Eifeligalehu"* in Amharic, did not appear to be indicating instructions and was therefore replaced with another term *"Eteyiqwotalehu"*, meaning "I ask you/ask of you". Besides, in all items, the endings of the questions were changed to *"eteyik****wotalehu****/Yad****irgu"***, which gives plural meanings when directly translated to the English language. Nevertheless, in the majority of Ethiopian cultures and also in the Amharic language, it is considered a sign of respect to older people.

Item 4 in RUDAS (judgment): The term "…busy street…" whose direct semantic equivalence in Amharic is *"*…*Yetechenaneke godana*…*"* did not clearly provide the sense of the question as it could also mean "streets where no cars are passing by" and it was therefore replaced by another term "…Yetechenaneke yemekina menged…" which means "…Busy car road/busy road…". In this similar question, the phrase "traffic lights" was considered a less familiar term, and the lights are less abundant in the country. Therefore, the translation of the term included the addition of more descriptive terms and was put as "Yemenged teqotatari yemebrat milikitoch", which meant "Lights used to control the traffic flow".

Further steps and procedures were undertaken to ensure the RUDAS tool and items were translated into the Amharic language with better quality and intelligibility. The final translated version of the tool was administered to an independent sample of 20 individuals within the centre who were divided into two halves and were interviewed by a well-trained data collector. The interviewer asked each item to the first half of the participants in a face-to-face manner. After answering each item, respondents were asked to elaborate and explain their understanding of the question and their answers. They were also asked to suggest any local word that would fit in a better way. Modifications were made to those items when the meaning of the items was not clear, when the respondents found it difficult to elaborate or when wrong conceptualization of the questions was identified from the participants’ responses. The items that needed modification were modified before administering the updated version to the second half of the participants similarly.

The following points were taken into consideration through the above procedures, i.e. the measurement aim of the questionnaires, the target population, the concepts that the questionnaire is intended to measure, and the interpretability of items.

RUDAS items were generally well understood, and the instructions were indicated to be precise. Seven of the first ten respondents indicated that the term "Tea" was not something to be purchased directly in a grocery store but rather the "Tea leaf" and that the word was indicated to be ambiguous. For this reason, it was replaced by another term, "Buna", meaning "Coffee", which is more popular and common in most parts of Ethiopia. The modified version was then administered to the remaining ten respondents, and no concern was raised. Before the data collection, a day-long training was provided for data collectors and supervisors on the instruments, ethical principles, and how they diagnose cases.

The assessment with the gold standard evaluation by psychiatry professionals and BSc nurses using RUDAS was interchanged for every 30 cases to avoid the order of tests effect.

### Data management and analysis

Every three days, the collected data was adequately assembled, reviewed, and checked for completeness and consistency by the PI. Only questionnaires that were complete were accepted.

The collected data were coded, and entered into Epi data entry V. 4.6.1 software and exported to Stata V.16 for analysis. After thorough data exploration, appropriate descriptive statics were determined and the results are reported with tables, texts, and figures.

Cronbach‘s alpha coefficient was used to measure internal consistency reliability. The intra-class correlation (Kappa) coefficient was used to determine inter-rater reliability.

The content validity of RUDAS was determined by calculating the Content Validity Index (CVI). A panel of experts with nine members was selected from senior professionals with knowledge and experience in the field of Psychiatry (1 expert with PhD in Psychiatry, 3 Psychiatrists, and 2 MSc in mental health), Epidemiology (1 expert with PhD in Epidemiology), and Neurology (2 Neurologists) to rate each item in the tool. Each panel member was asked to rate each item of the tools from 1–4 based on relevance and clarity. A score of 1 was deemed not relevant, 2—relevant but needs revision, 3—relevant with minor revision, and 4- relevant. The total number of experts who gave 3–4 (relevant) was divided by the total number of experts to calculate the I-CVI. S-CVI using the averaging approach (S-CVI/Ave) was computed by averaging the sum of the I-CVIs of the items of the tool to the total number of items. The definition and detailed procedures of calculating content validity indexes are described elsewhere and can be found in the literature [36].

Specificity, sensitivity, positive and negative predictive values, as well as positive and negative likelihood ratios, were calculated at several cutoff scores. Youden’s J index (sensitivity + specificity−1) was used to determine the optimal cutoff score with the best balance of sensitivity and specificity. Receivers operating curve (ROC) analysis with the corresponding Area Under Curve (AUC) was determined against the gold standard to determine accuracy. Pearson’s correlation coefficient was calculated to determine the correlation between screening tools (concurrent validity). A correlation coefficient of 0.6 or more was judged as indicating a strong association considering the unreliability associated with the scales due to the attenuation of validity coefficients [37].

Construct validity was determined by using the known group validity approach. An independent sample t-test was used to determine a significant mean difference in the test scores among the dementia and no dementia groups based on the gold standard assessment. Statistical significance was declared with P values < 0.05.

Multiple linear regression analysis was employed to determine the association of scores of RUDAS with different factors. Statistical significance was declared at P < 0.05. All the assumptions of multiple linear regression such as linear relationship, normality of residuals, multi-collinearity, and homoskasadicity were checked.

### Ethical approval and consent to participate

Ethical approval was obtained from the institutional ethical review board of Jimma University, Institute of Health, with the reference number of IRB000263/2012. Before data collection, written informed consent was obtained from the study participants after a detailed explanation of the purposes of the study. Confidentiality was maintained for the participants. All the necessary precautions were employed during the data collection process to prevent the spread of COVID-19 infection.

## Results

### Socio-demographic and clinical characteristics

A total of 116 respondents participated in the study with a response rate of 100%. The mean age of the participants was 69.87 ± 7.97 (Range; 60–94) and males comprised 51.72% (n = 60) of the participants. The majority, 48.30% (n = 56) of the respondents had attended no formal education and the mean years for formal education attended by the respondents was 4.90 ± 5.90 years. The mean RUDAS scores has showed a steady increase as the participants educational level increased with those who attended college and above scoring the highest mean RUDAS score (27 ±2.5) and those with no formal education scoring the lowest (18.21 ± 5.7). Regarding marital status, 42.24% (n = 49) of them were widowed/widower. Before their admission to the centre, 55.17% (n = 64) of the respondents had their residence in urban areas. The mean (SD) score on geriatric depression scale short form of the participants was 6.01 (3.59). Those with a diagnosis of dementia had a mean (SD) MMSE score of 15.36 (1.54) while those who didn’t have dementia had a mean (SD) MMSE score of 23.83 (1.31). Other sociodemographic and clinical characteristics of the respondents can be observed in Table 1.

**Table 1:** Socio demographic and clinical characteristics with mean scores on RUDAS of the study respondents.

Variable	Category	Frequency	Percent	Mean (SD) RUDAS
Age	60–64	34	29.31	24.12 (1.4)
	65–69	32	27.59	22.31 (1.8)
	70–74	27	23.28	17.11 (2.5)
	75 and above	23	19.82	21.69 (2.2)
Sex	Male	60	51.72	21.67 (5.7)
	Female	56	48.28	21.34 (5.9)
Ethnicity	Amhara	39	33.62	21.76 (5.7)
	Oromo	43	37.07	21.23 (6.5)
	Guragie	14	12.07	20.36 (4.6)
	Tigre	15	12.93	22.53 (4.3)
	Others*	5		22.00 (7.1)
Mother tongue	Amharic	42	36.21	22.02 (5.6)
	Affan Oromo	41	35.34	21.09 (6.7)
	Tigrigna	15	12.93	22.53 (4.3)
	Guragigna	13	11.21	19.77 (4.3)
	Others^#^	5		22 (7.1)
Religion	Orthodox	69	59.48	20.80 (5.9)
	Muslim	14	12.07	20 (6.2)
	Protestant	26	22.41	23.65 (4.9)
	Catholic	7	6.03	23.57 (5.4)
Educational status	No formal education	56	48.80	18.21 (5.7)
	Primary Education	32	27.59	23.19 (4.1)
	Secondary Education	13	11.21	25.23 (2.5)
	College and above	15	12.93	27 (2.5)
Marital Status	Never Married	24	20.69	22.29 (5.5)
	Married	33	28.45	24.42 (5.1)
	Divorced	10	8.62	19.6 (5.3)
	Widowed/Widower	49	42.24	19.55 (5.6)
Area of residence before admission to the center	Urban	64	55.17	22.53 (5.5)
	Rural	52	44.83	20.25 (5.9)
Diagnosis (DSM-5)	Dementia	39	33.62	16.84 (4.2)
	No Dementia	77	66.38	23.87 (5)
Depression (GDS)	No depression	44	37.93	24.70 (3.7)
	Mild to moderate depression	61	52.59	20.03 (5.6)
	Severe depression	11	9.48	16.91 (7.6)
Total		116	100	21.5 (5.7)

* Others = Sidama, Wolita, Kaffa.

^#^ Others = Sidamu, Wolitigna, Kaffigna.

### Reliability of RUDAS

RUDAS had a Cronbach’s alpha value of 0.73. None of the items in the tool have resulted in an increment of its alpha values when they were deleted. The inter-rater reliability findings indicate that RUDAS had an ICC value of 0.94 (95% CI: 0.82–0.98).

### Validity of RUDAS

#### Content validity

RUDAS had excellent Content validity index results. Only one item, i.e. Visuo-constructional drawing, had an item-level content validity index (I-CVI) of 80% with the rest scoring 100%. The scale level content validity index (S-CVI) of the tool was 96.7%.

#### Criterion related validity

**Fig 1:**
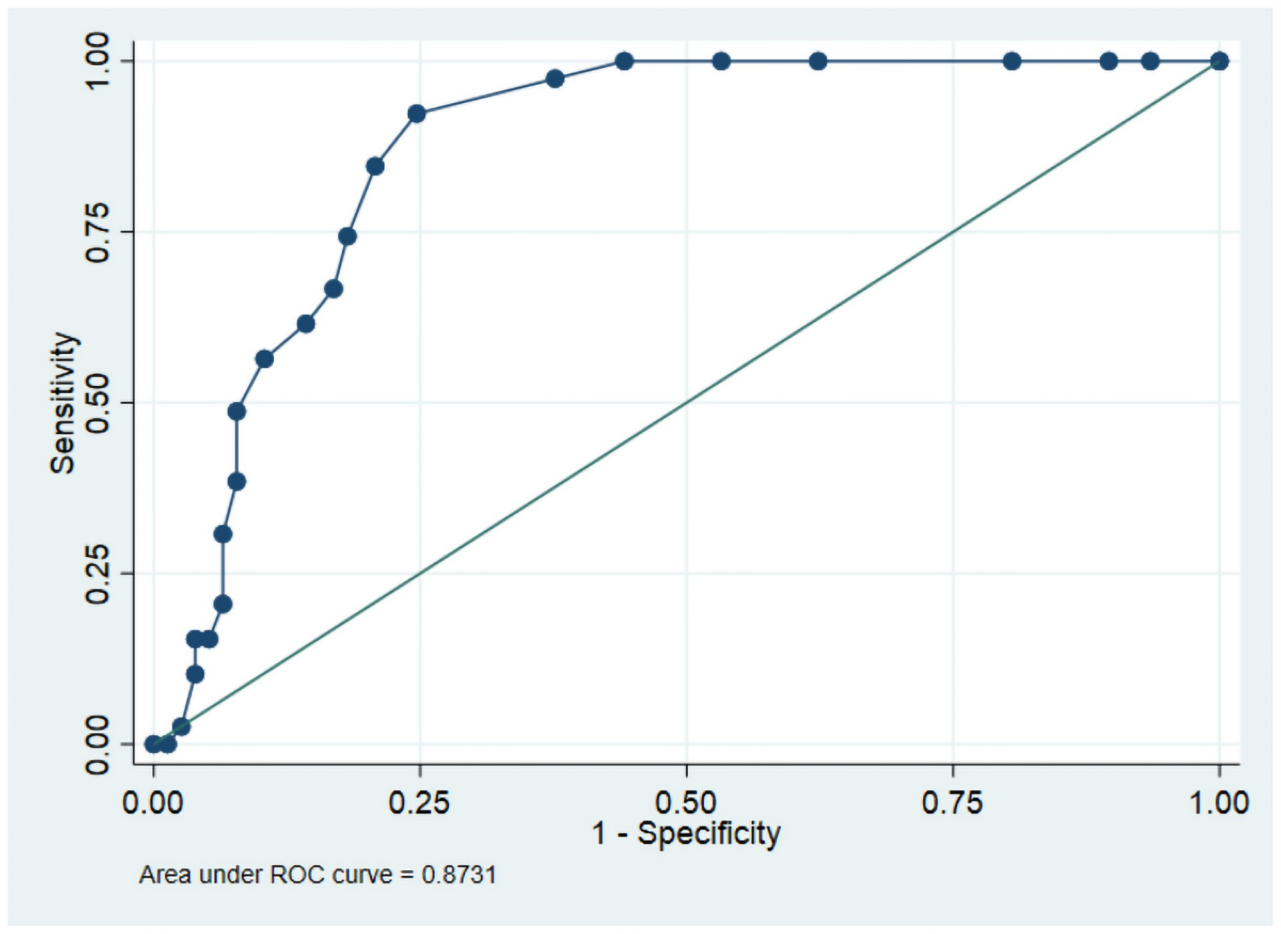
ROC curve of RUDAS for the identification of major neuro-cognitive disorder, 2020.

For the total respondents who participated in the study, the mean score of RUDAS was 21.5 ± 5.7. On average, the administration of RUDAS took 10 minutes. The area under the receiver’s operating curve for the identification of major neurocognitive disorder was AUC = 0.87 (95%CI: 0.81–0.93) (Fig 1).

At the recommended cutoff score of RUDAS, which was ≤23, it had excellent sensitivity (97.4%) but had lower specificity at 62.3%. The optimal cutoff score for the tool for to detect major neurocognitive disorder determined in our study based on the maximum Youden’s J index was scores less than or equal to 22.

At this cut point, the tool had a sensitivity value of 92.31% and a specificity value of 75.32% and it correctly classified the cases 81.03% of the time (Table 2). The LR+, LR-, PPV and NPV values of the tool at the specified optimal cutoff value were 3.74, 0.10, 65.5%, and 91.5%, respectively.

**Table 2:** Psychometric properties of RUDAS for the diagnosis of major neurocognitive disorder, 2020.

Cutoff (≤)	Sensitivity	Specificity	Correctly classified	Youden’s J index	LR+	LR-	PPV	NPV
19	66.67%	83.12%	77.59%	0.49	3.94	0.40	66.74%	83.10%
20	74.36%	81.82%	79.31%	0.56	4.08	0.31	67.42%	86.30%
21	84.62%	79.22%	81.03%	0.64	4.07	0.19	67.30%	91.00%
**22**	**92.31%**	**75.32%**	**81.03%**	**0.67**	**3.74**	**0.10**	**65.58%**	**95.10%**
23	97.44%	62.34%	74.14%	0.59	2.58	0.04	56.73%	98.00%
24	100%	55.84%	70.69%	0.56	2.26	0.00	53.41%	100%

#### Construct validity and concurrent validity

Independent sample t-test analysis revealed a statistically significant difference between the two diagnostic groups in their mean RUDAS scores (mean diff. 7.02, t = 7.7, p<0.001). Correlation analysis revealed that there is a statistically significant, positive correlation between the tool and the MMSE (r = 0.68, p<0.01).

#### Factors associated with the performance of the RUDAS

A single-year increase in attending formal education was associated with 0.312 points increase in RUDAS scores (β: 0.312 [95% CI: 0.147, 0.478], P<0.001). Having a diagnosis of dementia was associated with 5 points reduction in RUDAS scores (β: - 5.052 [95% CI: -6.819, -3.286], P<0.001) and a one-point increase in GDS scores resulted in 0.39 points decrement in RUDAS scores (β: -0.395 [95% CI: - 0.626, - 0.164], P = 0.001) (Table 3). The variables in the model explained 52% of the variation observed in the RUDAS scores (R^2^ = 0.56, Adjusted R^2^ = 0.52, F = 13.27, p<0.001).

**Table 3:** Multiple linear regression model results for factors associated with RUDAS scores, 2020.

Variable	Coef. (β)	Sig.	[95% Conf. Interval]	
			Upper Bound	Lower Bound
Age	-0.046	0.363	- 0.147	0.054
Years of Formal Education	0.312	**<0.001**	0.147	0.478
Mother Tongue^#^				
Affan Oromo	-1.669	0.066	-3.451	0.112
Tigrigna	0.282	0.820	-2.164	2.728
Guragigna	-0.368	0.782	-2.993	2.257
Others	-0.655	0.740	-4.555	3.244
Gender (Female)	0.905	0.264	- 0.692	2.501
Residence (Rural)	0.221	0.790	-1.421	1.863
Dementia (Yes)	- 5.052	**<0.001**	-6.819	-3.286
GDS total score	-0.395	**0.001**	- 0.626	- 0.164
__constant	27.389	0.000	19.447	35.331

* Dependent variable: RUDAS total score;

^#^ Amharic taken as a constant;

^##^ Sidamu, Wolitigna, Kaffigna

## Discussion

In this study, we validated and reported the psychometric properties and diagnostic accuracy of RUDAS to detect major neurocognitive disorder in Ethiopia. RUDAS showed very good internal consistency reliability with Cronbach’s alpha value of 0.73. In the current study, at an optimal cut point of ≤ 22, RUDAS has demonstrated an excellent ability to detect major neurocognitive disorder with an AUROC value of 0.87 and sensitivity and specificity values of 92% and 75%, respectively. The participants’ years of formal education showed a statistically significant positive association with RUDAS scores, whereas having dementia and the geriatric depression score results showed significant negative associations with total RUDAS scores.

Many validity studies in different settings and languages have also reported very good reliability measures for RUDAS. The initial development study by Storey et al. reported an inter-rater, and test-retest reliabilities of 0.99 and 0.98, respectively [23]. In addition to the above study, the reliability measures for RUDAS on the current study also corroborate with reports of the studies conducted in the Netherlands, Taiwan, Peru, and Nepal [32, 38–40]. These consistent reports demonstrate the tool’s ability to reliably and consistently measure the cognitive status of individuals.

The initial validation study of RUDAS by Storey et al. in 2004 reported an area under the ROC curve of 0.95. At an optimal cutoff score of 23, the sensitivity was 89% and the specificity was 98% [23]. Adding to the studies assessing the performance of RUDAS in communities with low and middle socioeconomic status, another one conducted in Taiwan reported that RUDAS had an AUC of 0.87, sensitivity 76%, 83% PPV, a specificity of 81%, and 91% NPV with a cut point of 22 [32].

The optimal cutoff value in our study was one point lower than the one recommended by the tool developers and some other studies conducted in high-income countries [23, 30]. The observed variation may be due to the high socioeconomic status and a better level of education of the participants in those studies. This was further supported as the tool has a similar cutoff point and AUC value as another study conducted in Taiwan, with a sample of low-education and low-income background [32]. The similarity in the findings indicates that RUDAS can be successfully applied in low and middle-income countries to screen for cognitive impairment.

RUDAS has been reported to be a tailor-made tool for communities with diverse backgrounds. The initial validation study reported that factors such as gender, years of education, cultural background, and preferred language were not associated while age was [23]. Several other studies conducted to validate the tool reported similar findings [12, 28, 39]. However, controversies exist regarding the association of education with the performance of the test. Some report the relative freeness of the tool from the effect of education [38, 40, 41] while others indicated an association [30, 42]. In our study, confirming the findings of some of the studies, years of education had a significant association. However, the excellent performance of the test in the current study population with low mean years of education, i.e. five years, indicates that it can be applied effectively in such communities.

The findings of this study overall indicate that the tool is a practical, valid, and reliable instrument for screening major neurocognitive disorder and assessing individual’s cognitive status. In resource-limited settings like Ethiopia, having validated brief screening tools will help in the early identification of cognitive impairments, which will, in turn, lead to early interventions aimed at stopping or slowing the progression of the disorder.

The excellent performance of the tool in a sample with a low mean level of education and a high cultural diversity indicates the applicability of the tool in such populations. The short time taken to administer the tool and also the administration of the tool by non-psychiatry professionals provides the advantage of applicability of the tool in busy outpatient setups and by professionals outside of mental health practice. As far as the researcher’s best knowledge, this study is the first to validate RUDAS in the continent of Africa to this date.

### Limitations

The variability of the Amharic language across different regions of the country in wording and cultural difference might require caution in using this version of the tool in different areas of the country and the need for further validation studies in the other languages. The current study also did not assess the test-retest reliability of RUDAS. Test-retest reliability is an essential measure of the tool’s stability and ability to consistently screen for major neurocognitive disorder over time.

## Conclusions and recommendations

The Rowlands universal dementia assessment scale has been demonstrated to be a valid and reliable cognitive assessment instrument and can be incorporated in clinical and research practices in developing countries. Researchers in the area can also conduct further validation studies to assess the applicability of the tool in other languages and communities. Future studies should also assess the test-retest reliability of the tool.

## Abbreviations

AUC: Area Under Curve
CVI: Content Validity Index
DSM: Diagnostic and Statistical Manual
GDS: Geriatric Depression Scale
HIV: Humane Immune Virus
LMIC: Low and Middle Income
MMSE: Mini Mental State Examination
ROC: Receivers Operating Curve
RUDAS: Rowland’s Universal Dementia Assessment Scale

## References

[1] United Nations. World Population Prospects 2019 [Internet]. Department of Economic and Social Affairs. World Population Prospects 2019. 2019. 49–78 p. http://www.ncbi.nlm.nih.gov/pubmed/12283219.

[2] United Nations Department of Economic and Social Affairs ǀ Population Division. World Population Ageing 2013. Vol. 4, World Population Ageing. New York; 2013.

[3] WHO. World Report on Aging and Health. Vol. 1, World Health Organization. 2015.

[4] Prince M. The Global Impact of Dementia; An Analysis of Prevalence, Incidence, Cost And Trends [Internet]. World Alzheimer Report. London; 2015. https://www.alz.co.uk/research/WorldAlzheimerReport2015.pdf.

[5] Sadock BJ, Sadock VA, Ruiz P. KAPLAN & SADOCK’S COMPREHENSIVE TEXTBOOK OF PSYCHIATRY VOLUME I / I I, 10th ed. 2017.

[6] Livingston G, Sommerlad A, Orgeta V, Costafreda SG, Huntley J, Ames D, et al. Dementia prevention, intervention, and care. Lancet. 2017;390(10113):2673–734. doi: 10.1016/S0140-6736(17)31363-6 28735855

[7] American Psychiatric Association. DIAGNOSTIC AND STATISTICAL MANUAL OF MENTAL DISORDERS FIFTH EDI T ION, DSM-5TM. 2013.

[8] Prince M, Guerchet M, Prina M. The Global Impact of Dementia 2013–2050 Policy Brief for Heads of Government. Policy Br Heads Gov. 2013;1–8.

[9] Patterson C. World Alzheimer Report 2018 The state of the art of dementia research: New frontiers. Alzheimer’s Disease International. 2018.

[10] Prince M, Ali GC, Guerchet M, Prina AM, Albanese E, Wu YT. Recent global trends in the prevalence and incidence of dementia, and survival with dementia. Alzheimer’s Res Ther [Internet]. 2016;8(1). Available from: 10.1186/s13195-016-0188-8. PMC4967299 27473681

[11] Monroe T, Carter M. Using the Folstein Mini Mental State Exam (MMSE) to explore methodological issues in cognitive aging research. Eur J Ageing. 2012;9:265–74. doi: 10.1007/s10433-012-0234-8 28804426 PMC5547414

[12] Matiás-Guiu JA, Valles-Salgado M, Rognoni T, Hamre-Gil F, Moreno-Ramos T, Matiás-Guiu J. Comparative Diagnostic Accuracy of the ACE-III, MIS, MMSE, MoCA, and RUDAS for Screening of Alzheimer Disease. Dement Geriatr Cogn Disord. 2017;43(5–6):237–46. doi: 10.1159/000469658 28384640

[13] Prince M, Bryce R, Ferri C. The benefits of early diagnosis and intervention [Internet]. World Alzheimer Report 2011. London; 2011. https://www.alz.co.uk/research/WorldAlzheimerReport2011.pdf.

[14] Brodaty H, Low L, Hons BSP, Burns K, Hons BP. What Is the Best Dementia Screening Instrument for General Practitioners to Use? Am J Geriatr Psychiatry [Internet]. 2006;14(5):391–400. Available from: 10.1097/01.JGP.0000216181.20416.b2 16670243

[15] Folstein MF, Folstein SE. “Mini Mental state” A practical method for grading the cognitive state of patients for the clinician. J Psychiatr Res. 1975;12:189–98. doi: 10.1016/0022-3956(75)90026-6 1202204

[16] Ibrahim SA. Critical Perspectives on Racial and Ethnic Differences in Health in Late Life. Vol. 142, Annals of Internal Medicine. 2005. 80 p.

[17] Escobar JI et al. Use of the Mini-mental State Examination (MMSE) in a community population of mixed ethnicity. Cultural and linguistic artifacts. J ofNervous Andm Dis. 1986;174:607–614. 10.1097/00005053-198610000-00005 3760851

[18] Limpawattana P, Tiamkao S, Sawanyawisuth K, Thinkhamrop B. Can Rowland Universal Dementia Assessment Scale (RUDAS) Replace Mini-Mental State Examination (MMSE) for Dementia Screening in a Thai Geriatric Outpatient Setting? Am J Alzheimer’s Dis Other Dementias. 2012;27(4):254–9. 10.1177/1533317512447886 PMC10697337 22615482

[19] Basic D, Rowland JT, Conforti DA, Vrantsidis F, Hill K, LoGiudice D, et al. The validity of the Rowland Universal Dementia Assessment Scale (RUDAS) in a multicultural cohort of community-dwelling older persons with early dementia. Alzheimer Dis Assoc Disord. 2009;23(2):124–9. doi: 10.1097/wad.0b013e31818ecc98 19484915

[20] Gugssa SA, Davey G, Ejigu AA, Metaferia GZ, Medhin G, Kelkile TS. Population norms for the mini-mental state examination in ethiopia. Ethiop Med J. 2011;49(3):239–47. 21991757

[21] Christine S, Alemayehu N, Markos T, Herbert S. NEUROPSYCHOLOGICAL DIAGNOSTICS IN ETHIOPIA—CHALLENGES AND CHANCES AMONG CONSIDERATIONS REGARDING DIFFERENTIAL DIAGNOSIS (LITERATURE OVERVIEW). African J Neurol Sci. 2014;33(1):84–93.

[22] UNESCO Institute for Statistics. Ethiopia | UNESCO UIS [Internet]. UNESCO. 2017 [cited 2019 Dec 25]. http://uis.unesco.org/en/country/et.

[23] Storey JE, Rowland JTJ, Conforti DA, Dickson HG. The Rowland Universal Dementia Assessment Scale (RUDAS): A multicultural cognitive assessment scale. Int Psychogeriatrics. 2004;16(1):13–31. doi: 10.1017/s1041610204000043 15190994

[24] Cheung G, Clugston A, Croucher M, Malone D, Mau E, Sims A, et al. Performance of three cognitive screening tools in a sample of older New Zealanders. Int Psychogeriatrics. 2015;27(6):981–9. 10.1017/S1041610214002889 25603424

[25] Pang J, Yu H, Pearson K, Lynch P, Fong C. Comparison of the MMSE and RUDAS cognitive screening tools in an elderly inpatient population in everyday clinical use. Intern Med J. 2009;39(6):411–4. doi: 10.1111/j.1445-5994.2009.01918.x 19580621

[26] Komalasari R, Chang HC, Traynor V. A review of the Rowland Universal Dementia Assessment Scale. Dementia. 2019;18(7–8):3143–58. doi: 10.1177/1471301218820228 30606042

[27] Naqvi RM, Haider S, Tomlinson G, Alibhai S. Cognitive assessments in multicultural populations using the rowland universal dementia assessment scale: A systematic review and meta-analysis. CMAJ [Internet]. 2015 Mar 17;187(5):E169–76. Available from: http://www.cmaj.ca/lookup/doi/10.1503/cmaj.140802 25691786 10.1503/cmaj.140802 PMC4361127

[28] Basic D, Khoo A, Conforti D, Rowland J, Vrantsidis F, Logiudice D, et al. Rowland universal dementia assessment scale, mini-mental state examination and general practitioner assessment of cognition in a multicultural cohort of community-dwelling older persons with early dementia. Aust Psychol. 2009;44(1):40–53.

[29] De Araujo NB, Nielsen TR, Engedal K, Barca ML, Coutinho ES, Laks J. Diagnosing dementia in lower educated older persons: Validation of a Brazilian portuguese version of the Rowland universal dementia assessment scale (RUDAS). Rev Bras Psiquiatr. 2018;40(3):264–9. doi: 10.1590/1516-4446-2017-2284 29451587 PMC6899391

[30] Nielsen TR, Segers K, Vanderaspoilden V, Bekkhus-Wetterberg P, Bjørkløf GH, Beinhoff U, et al. Validation of the Rowland Universal Dementia Assessment Scale (RUDAS) in a multicultural sample across five Western European countries: Diagnostic accuracy and normative data. Int Psychogeriatrics. 2019;31(2):287–96. 10.1017/S1041610218000832 30017010

[31] Rowland J, Conforti D, Basic D, Vrantsidis F, Hill K, LoGiudice D, et al. A study to validate the Rowland Universal Dementia Assessment Scale (RUDAS) in two populations outside the Sydney South West Area Health Service A report to and funded by: 2007;1–40.

[32] Chen CW, Chu H, Tsai CF, Yang HL, Tsai JC, Chung MH, et al. The reliability, validity, sensitivity, specificity and predictive values of the Chinese version of the Rowland Universal Dementia Assessment Scale. J Clin Nurs. 2015;24(21–22):3118–28. doi: 10.1111/jocn.12941 26259826

[33] Hanley JA, Mcneil J, Ph D. A Method of Comparing the Areas under Receiver Characteristic Operating Curves Derived from the Same Cases ‘. Radiology. 1983;148:839–43. doi: 10.1148/radiology.148.3.6878708 6878708

[34] Yesavage JA, Brink TL, Rose TL, Lum O, Huang V, Adey M, et al. Development and validation of a geriatric depression screening scale: A preliminary report. J Psychiatr Res. 1982;17(1):37–49. doi: 10.1016/0022-3956(82)90033-4 7183759

[35] Stone LE, Granier KL, Segal DL. Geriatric Depression Scale. Colorado Springs; 2019.

[36] Zamanzadeh V, Ghahramanian A, Rassouli M, Abbaszadeh A, Alavi-M H. Design and Implementation Content Validity Study: Development of an instrument for measuring Patient-Centered Communication. 2015;4(5):165–78. Available from: 10.15171/jcs.2015.017. PMC4484991 26161370

[37] Mcdowell I. Measuring Health: A Guide to Rating Scales and Questionnaires. Third Edit. New York: Oxford University Press, Inc; 2006.

[38] Goudsmit M, Van Campen J, Schilt T, Hinnen C, Franzen S, Schmand B. One Size Does Not Fit All: Comparative Diagnostic Accuracy of the Rowland Universal Dementia Assessment Scale and the Mini Mental State Examination in a Memory Clinic Population with Very Low Education. Dement Geriatr Cogn Dis Extra. 2018;8(2):290–305. doi: 10.1159/000490174 30323830 PMC6180264

[39] Custodio N, Montesinos R, Lira D, Herrera-Perez E, Chavez K, Hernandez-Córdova G, et al. Validation of the RUDAS in Patients With a Middle-Level Education in Lima, Peru. Am J Alzheimers Dis Other Demen. 2019;34(7–8):513–22. doi: 10.1177/1533317519869709 31422688 PMC10653366

[40] Nepal GM, Shrestha A, Acharya R. Translation and cross-cultural adaptation of the Nepali version of the Rowland universal dementia assessment scale (RUDAS). J Patient-Reported Outcomes. 2019;3(1). 10.1186/s41687-019-0132-3 PMC6639471 31321572

[41] Mateos-Álvarez R, Ramos-Ríos R, López-Moríñigo JD. Comparative analysis between the MMSE and the RUDAS for dementia screening in low educated people in a Spanish psychogeriatric clinic. Eur J Psychiatry. 2017;31(3):119–26.

[42] Iype T, Ajitha BK, Antony P, Ajeeth NB, Job S, Shaji KS. Usefulness of the Rowland Universal Dementia Assessment Scale in South India. J Neurol Neurosurg Psychiatry. 2006;77(4):513–4. doi: 10.1136/jnnp.2005.069005 16543532 PMC2077504

